# Protein domain recurrence and order can enhance prediction of protein functions

**DOI:** 10.1093/bioinformatics/bts398

**Published:** 2012-09-03

**Authors:** Mario Abdel Messih, Meghana Chitale, Vladimir B. Bajic, Daisuke Kihara, Xin Gao

**Affiliations:** ^1^Mathematical and Computer Sciences and Engineering Division; ^2^Computational Bioscience Research Center, King Abdullah University of Science and Technology (KAUST), Thuwal, 23955-6900, Saudi Arabia; ^3^Department of Computer Science; ^4^Department of Biological Sciences, College of Science; ^5^Markey Center for Structural Biology, Purdue University, West Lafayette, Indiana, USA.

## Abstract

**Motivation:** Burgeoning sequencing technologies have generated massive amounts of genomic and proteomic data. Annotating the functions of proteins identified in this data has become a big and crucial problem. Various computational methods have been developed to infer the protein functions based on either the sequences or domains of proteins. The existing methods, however, ignore the recurrence and the order of the protein domains in this function inference.

**Results:** We developed two new methods to infer protein functions based on protein domain recurrence and domain order. Our first method, DRDO, calculates the posterior probability of the Gene Ontology terms based on domain recurrence and domain order information, whereas our second method, DRDO-NB, relies on the naïve Bayes methodology using the same domain architecture information. Our large-scale benchmark comparisons show strong improvements in the accuracy of the protein function inference achieved by our new methods, demonstrating that domain recurrence and order can provide important information for inference of protein functions.

**Availability:** The new models are provided as open source programs at http://sfb.kaust.edu.sa/Pages/Software.aspx.

**Contact:** dkihara@cs.purdue.edu, xin.gao@kaust.edu.sa

**Supplementary information:** Supplementary data are available at *Bioinformatics Online*.

## 1 INTRODUCTION

Proteins play vital roles in biological systems. Understanding their functions is one of the most important problems in biology today. Due to rapid advances in genomic sequencing techniques and computational gene identification, the number of explored protein sequences has increased dramatically. A number of experimental methods has been developed to predict the functions of proteins (Hawkins and Kihara, 2007; Pandey *et al*., 2006). These experimental methods, however, cover only a limited number of experimental conditions and have limited protein coverage. In addition, these methods cannot follow the exponential increase in the number of newly discovered protein sequences or their variants, caused by improved sequencing technologies.

A number of protein databases has been compiled such as UniProt (Wu *et al*., 2006), PDB (Deshpande *et al*., 2005) and CATH (Pearl *et al*., 2005). These databases, along with controlled function vocabularies like Gene Ontology (GO), have made computational annotation of protein functions easier. Various computational techniques have been applied to predict protein functions based on different features of protein sequences, such as protein functional sites and domains (Forslund and Sonnhammer, 2008; Jung and Thon, 2006; Vogel *et al*., 2004), sequence similarity (Chitale *et al*., 2009; Khan *et al*., 2003; Martin *et al*., 2004; Sael *et al*., 2012; Vinayagam *et al*., 2004) and gene expression patterns (Pavlidis *et al*., 2002). A comprehensive summary of the existing techniques can be found in a number of reviews (Hawkins and Kihara, 2007; Pandey *et al*., 2006; Rentzsch and Orengo, 2009).

The classical approach to the annotation of protein functions is based on sequence similarity using BLAST (Altschul *et al*., 1997) or similar programs. Other variants of this classical approach include building phylogenetic trees to infer protein functions based on proteins from the same subfamily (Engelhardt *et al*., 2005; Krishnamurthy *et al*., 2007). On the other hand, machine learning methods have been extensively applied to protein function annotation, including support vector machines (Dobson *et al*., 2003; Tan *et al*., 2009), naïve Bayes (NB) (Forslund and Sonnhammer, 2008), and decision trees to represent the GO annotation hierarchy (Hayete and Bienkowska, 2005; Ivanoska *et al*., 2010). There are also significant efforts in developing protein tertiary structure-based function prediction methods that either consider global (Orengo *et al*., 1994; Sael *et al*., 2008) or local structural similarity (Chikhi *et al*., 2010; Sael *et al*., 2012).

Generally, it is believed that functions of a protein are carried out via protein domains, which are protein's functional or structural units. Hence, it is intuitive that protein functions can be inferred from the architecture of the domains. This logic has led to development of models to predict protein functions from domain information without regard to the protein amino acid sequence. Domain context information has been applied to automatic protein function prediction either implicitly or explicitly (Beaussart *et al*., 2007; Coin *et al*., 2003; Forslund and Sonnhammer, 2008; Hayete and Bienkowska, 2005; Mulder *et al*., 2007; Silvescu *et al*., 2004; Song *et al*., 2007).

Silvescu *et al*. (2004) proposed several naïve Bayes models for protein function annotation. Instead of using domains as the units in their models, they selected *k* consecutive amino acids, i.e. *k*-grams, as the units. The dependency between the *k*-grams was modeled by the naïve Bayes approach. Hayete and Bienkowska (2005) applied the decision tree technique to assign functions to domains. Pfam2GO was proposed by Mulder *et al*. (2007). They assigned GO terms to the individual domains by sequentially mapping the InterPro domains (Apweiler *et al*., 2001) to the Pfam domains. However, Pfam2GO suffered from low sensitivity. Song *et al*. (2007) proposed algorithms for protein alignment based on domain content. Their method was analogous to the idea of homology search for protein sequences.

Forslund and Sonnhammer (2008) proposed two protein function prediction methods using domain content. The method that performed better of the two was the probabilistic model, which was more accurate (higher precision) than the conventional BLAST-based method, although the sensitivity of the model was lower, suggesting that the model was able to predict highly reliable function annotations, but with a trade-off for lower coverage. High precision is preferred in automatic annotation of protein functions, however.

We hypothesize that protein functions are not determined only by the dependency of the protein on the presence of domains, but also by the recurrence and order of the domains. This information has not been utilized in the existing protein function predictors, to the best of our knowledge. To evaluate our hypothesis, we developed two new methods that use such information explicitly, one that determines posterior probability of the GO terms based on domain architecture (referred to as the DRDO model), and the other that relies on the naïve Bayes methodology (referred to as the DRDO-NB model).

We tested our newly developed models on several large-scale benchmark datasets. The test results show that our models outperform to a great extent a number of state-of-the-art predictors of protein functions on the curated datasets. This supports our hypothesis of the importance of the domain recurrence and order information in inference of protein functions.

## 2 ILLUSTRATIVE SUPPORTING EXAMPLES

Here, we present two examples that illustrate the importance of the domain recurrence and domain order in function determination. Both of these examples are based on the violation of the Koide assumption (Koide, 2009). Consider a protein with a domain architecture A*B*B*A*C. The existing models assume that the protein will have the same functions as proteins with domain architectures A*B*C or B*C*A. Although there is evidences that some domain rearrangements do not necessarily alter functions (Koide, 2009), many of such domains do affect protein functions. The following two examples illustrate our point.

**Fig. 1.**
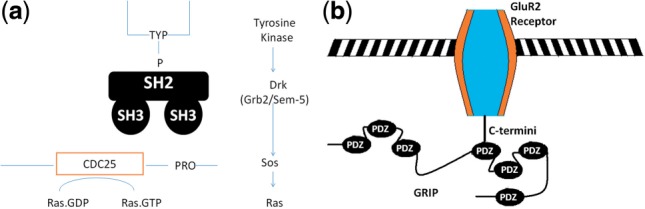
Two examples that demonstrate the importance of domain recurrence and domain order. **(a)** Mechanism for DRK to couple activated receptor tyrosine kinases to Sos. DRK binds to the activated receptor tyrosine kinases through its SH2 domain, and binds to Sos through its two SH3 domains. Two SH3 domains are required to achieve the affinity for binding. **(b)** Mechanism of the interaction between GRIP and the AMPA receptor. The fourth and fifth PDZ domains of GRIP bind to the C-terminus of the GluR2 receptor of AMPA. Syntenin, which consists of two PDZ domains, does not bind to AMPA

1. An adaptor protein DRK (downstream of receptor kinase) is known to play an important role in sevenless receptor signaling in *Drosophila* (Le and Simon, 1998; Moressis *et al*., 2009; Olivier *et al*., 1993; Simon *et al*., 1993). DRK contains one SH2 domain and two flanking SH3 domains. DRK binds to the activated receptor tyrosine kinases through its SH2 domain, and it also binds to the C-terminal tail of Sos, a Ras guanine nucleotide-releasing protein that is required for sevenless receptor signaling, through its two SH3 domains, as shown in Figure 1(a). Previous studies demonstrated that both of SH3 domains are required to achieve binding affinity (Olivier *et al*., 1993; Simon *et al*., 1993). This suggests that information on both domain order and recurrence is essential in determining the functions of DRK. Otherwise, a protein that contains only one SH2 domain and one SH3 domain or a protein that contains two consecutive SH3 domains and one SH2 domain should perform the same functions in sevenless receptor signaling, which has been found not to be the case as described below.
  To further verify this observation, we checked the SH2-and SH3- containing proteins in UniProtKB. DRK has an accession number *Q08012*. *A2AVZ2*, a protein, contains one SH3 domain followed by one SH2 domain, whereas *Q920I1*, another protein, contains one SH2 domain followed by one SH3 domain. Neither *A2AVZ2* nor *Q920I1* has the GO term ‘GO:0045500: sevenless signaling pathway’, which belongs to DRK. The pairwise molecular function (MF) similarity when measured by simGIC (Pesquita *et al*., 2009) is 56, 18 and 21% for *Q08012* versus *A2AVZ2*, *Q08012* versus *Q920I1*, and *Q920I1* versus *A2AVZ2*, respectively. The detailed lists of GO term for the three proteins can be found in Supplementary Materials.
2. The PDZ domain is one of the most commonly observed structural domains found in signaling proteins from bacteria to humans (Nourry *et al*., 2003). The glutamate receptor interacting protein (GRIP) is a synaptic PDZ domain-containing protein that consists of seven PDZ domains (Dong *et al*., 1997). GRIP was found to interact with the C-terminus of the *α*-amino-3-hydroxy-5-methyl-4-isoxazolepropionic acid (AMPA) receptor (Dong *et al*., 1997; Lu and Ziff, 2005; Pawson and Nash, 2003), which is a transmembrane receptor for glutamate that plays an important role in long-term potentiation in the central nervous system and also in learning and memory. AMPA contains four subunits, i.e. GluR1-4 receptors. The fourth and fifth PDZ domains of GRIP interact with the C-terminus of GluR2 Figure 1b, which makes GRIP an adapter protein that links the AMPA receptor to other proteins. However, many of the other PDZ domain-containing proteins, such as syntenin, which consists of two PDZ domains, do not interact with the AMPA receptor (Nourry *et al*., 2003). This suggests that domain recurrence information is important to determining protein functions.
  To further verify this observation, we checked GRIP and syntenin in UniProtKB. GRIP's accession number is *P97879* and syntenin's is *O08992*. Syntenin does not have the GO term ‘GO:0007399 nervous system development’, which belongs to GRIP. The pairwise MF similarity when measured by simGIC is 5.7%. The detailed lists of GO term for the two proteins can be found in Supplementary Materials.

## 3 METHODS

The basic idea of our methods is that it is possible to predict relatively accurately the GO terms associated to a protein with *M* domains (unique or repeated or combination thereof) from the GO terms of proteins that are associated with subsets of these domains up to *M* −1 domains. Thus, in order to predict the function, i.e. GO terms, of a query protein that has *M* domains, we use the GO terms for all proteins that have up to *M* −1 of these domains and have GO annotation. Given a query protein, the domains are assigned to it based on information from the SwissPfam database (Sonnhammer *et al*., 1997), which provides the order of the domains present in this protein sequence. The GO terms for each domain or each subset of the domains are then extracted from the UniProtKB database. The goal is to predict the probability for each GO term among some 30,000 to be associated with the query protein. Here we propose two models to achieve this goal.

### 3.1 DRDO: a new probabilistic model

Given a domain set, *D*, and a GO term, *G*. According to the Bayes rule, the conditional probability of *G* given *D* can be calculated as
(1)
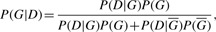

where *P*(·) denotes probability and 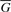 denotes the cases where the protein does not possess function *G*. Equation (1) can be rewritten as
(2)
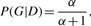

where 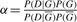. This conditional probability represents the posterior probability of a specific set of GO-terms *G* given the domain set *D*. By using the conditional independence assumption, which says that the distinct sets in which *P*(*D*|*G*) and 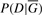 significantly differ occur independently, the odds ratio *α* can be estimated as
(3)
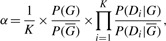

where the *D_i_*'s are the subsets of *D* that contain sequentially consecutive domains, and *K* represents the number of such subsets *D_i_*, such that *K* = *M* (*M* + 1)/2–1, where *M* is the number of domains in *D*. All the probabilities can be estimated by maximum likelihood estimation, i.e. counting the frequency in the training set. In contrast to the cross-validation training procedure for traditional machine learning methods, the training of our model follows that of Forslund and Sonnhammer (2008), in an incremental manner. That is, to predict functions for a protein with *M* domains, proteins with up to *M* – 1 domains are used as the training set. For instance, given a query protein with three domains, GO terms for each single domain are extracted from the UniProtKB database. Then the GO terms for each subset of two consecutive domains are predicted by using GO terms for single domains according to Equation (3). The GO terms for the query protein are then predicted by using GO terms for the single domains and the subsets of size two.

Note that our model generalizes and extends a related model proposed in Forslund and Sonnhammer (2008) (referred to as ‘FS model’ in the paper). In the FS model, both the domain recurrence and domain order are ignored when considering the protein domain architecture, *D*. That is, the domain set in the FS model is actually the unordered set of distinct domains in *D*. Therefore, for a domain set *D* = A*B*B*A*C, the corresponding *D_i_* subsets are {*A*}, {*B*}, {*C*}, {*AB*}, {*AC*}, {*BC*} and {*ABC*}.

The FS model has two drawbacks. First, the model does not consider the order of the domains. That is, a protein with a domain architecture A*B is assumed to have the same functions as another protein with the domain architecture B*A. This assumption is not always true as shown by the first example presented in Section 2. Second, the FS model ignores the recurrence of the same domain. That is, a protein with domain architecture A*A*A is assumed to have the same functions as proteins with domain architectures A*A or *A*. This assumption, again, is not always true according to both examples presented in Section 2.

It is clear that the main source of both of these drawbacks is the construction of the domain set, *D*. According to the FS model, *D* consists only of distinct domains. We can therefore preserve the domain recurrence and order information relatively well by defining *D* as the original set of all the domains in the protein and by defining the remaining orders of the domains when enumerating the *D_i_* subsets. That is, when we enumerate the subsets, we require each subset to contain only the sequentially neighboring domains on the protein. Therefore, given a protein with domain architecture *D* = A*B*B*A*C, the corresponding subsets are {*A*}, {*B*}, {*B*}, {*A*}, {*C*}, {*AB*}, {*BB*}, {*BA*}, {*AC*}, {*ABB*}, {*BBA*}, {*BAC*}, {*ABBA*} and {*BBAC*} (see the difference in subset construction presented above).

Our hypothesis raises two intuitive concerns. The first is that if the original domain set is used for enumeration, there will be many recurring *D_i_*'s because a domain can appear several times in a protein. Therefore, the conditional independence assumption may not hold. In fact, this is also an issue in the FS model. Even if only the distinct domains are used, the subsets are still not conditionally independent because they share common pairwise domains. Forslund and Sonnhammer tried different methods to reduce the Bayesian naïvete in their model. The best way was to normalize the odds ratio *α* by the size of the domain set, *K*, as shown in Equation (3). Furthermore, the naïve Bayes model is known to be tolerant to some degree of conditional dependence. Therefore, we also use the normalized version of *α* as shown in Equation (3) in our model.

**Fig. 2.**
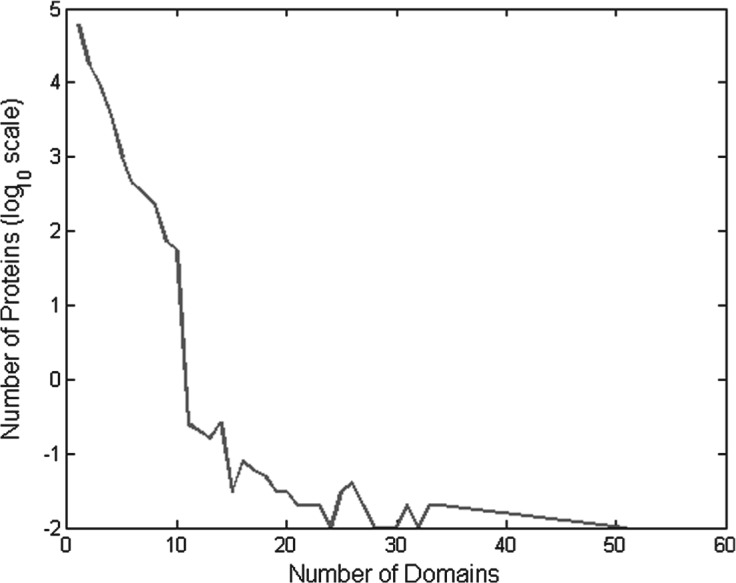
Distribution of the number of domains contained in the proteins in the UniprotKB/TrEMBL 2011 database. The number of proteins is shown in the log scale of base 10

The second concern is that since we enumerate all the subsets that contain the sequentially neighboring domains, of the original domain set, the number of subsets might be too large. In fact, the number of subsets enumerated by Forslund and Sonnhammer (2008) is 2*^N^* – 1, where *N* is the number of distinct domains in the protein. For our case, because we only consider the sequentially neighboring domains, the number of subsets is *M* (*M* +1)*/*2–1, where *M* is the total number of domains in the protein. As shown in Figure 2, the largest number of domains for currently known proteins is below 60, for which the number of subsets is approximately the number when *N* = 11 in the FS model. Further details about the practical runtime are discussed in Supplementary Materials.

### 3.2 DRDO-NB: a new naïve Bayes model

**Fig. 3.**
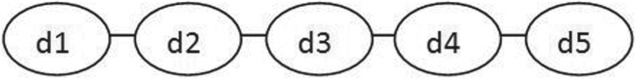
An example of a protein that has five domains with *k* = 2

We start first by illustrating our naïve Bayes model with pairwise dependency (*k* = 2) as shown in Figure 3. Under the assumption of pairwise dependency, the joint probability of the five domains is
(4)
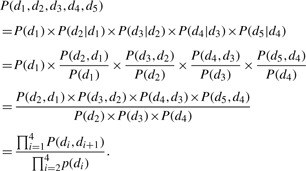

Similarly, we can generalize this rule for any protein that has *M* domains and dependency *k* as
(5)
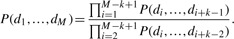


Therefore, we can formulate the function annotation problem as a classification problem, where the classification rule is
(6)
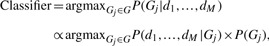

where *G* represents all the GO terms and *G_j_* denotes any single GO term. According to Equation (4), we have
(7)
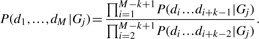


Furthermore we use the Laplace's correction to avoid zero probabilities and get a mild regularization effect. The basic idea of Laplace's correction is that we initialize the counts with 1 rather than 0, then add an appropriate count to the denominator to ensure that the probabilities sum up to 1.

We calculate Equation (7) for each *k* = 1,...,*M* −1, such that we can include all possible dependency combinations that encode domain recurrence and order information. We then compute the weighted average of the conditional probabilities and estimate the conditional probability for a specific *G_j_* given the domains of the protein, according to Equation (6). This procedure is repeated for each GO term and those with the largest probabilities are selected. In practice, this procedure can be efficiently improved as described in Supplementary Materials.

Contrary to Silvescu *et al*. (2004), which used amino acid sequence in building naïve Bayes models, we are exploiting information from protein domain recurrence and order. The models of Silvescu *et al*., 2004 suffer from a drawback that for a large protein, the number of features their models need is prohibitively large, which is not the case for our model.

### 3.3 Datasets

To evaluate the improvement from using domain recurrence and order information, two large-scale benchmark sets are selected. The first dataset is the same one used by Forslund and Sonnhammer (2008). This dataset consists of two different subsets. The first subset is a combination of the UniRef50 non-redundant dataset that has gene ontology annotations according to the Gene Ontology Annotation (GOA) database and whose proteins are present in the UniProt database. Since UniRef50 is non-redundant, function information is not taken from closely similar sequences in prediction for a query. The SwissPfam database was used to search the accession number of each protein and to determine the detailed domain architecture of the proteins. In this article, we call this subset ‘all’. The second subset includes only curated annotations which result from excluding any GOA with evidence code IEA (Inferred from Electronic Annotation) from the ‘all’ datasets. We call this subset ‘curated annotations only’.

We further selected a more up-to-date database, UniProtKB. The UniProtKB contains two subsets. The first is UniProtKB/Swiss-Prot, which contains the manual annotations. The second is UniProtKB/TrEMBL, which contains high-quality records from automatic annotation classification. Both UniProtKB/Swiss-Prot and UniProtKB/TrEMBL databases were downloaded on August 12, 2011. The domain architectures were extracted from the SwissPfam database.

## 4 RESULTS

Here, we compare the DRDO and DRDO-NB models with the FS model and several other state-of-the-art protein function predictors. The naïve Bayes model proposed in Silvescu *et al*. (2004) is not compared here because it is not publicly available. The results from the tests with the two datasets are discussed in this section. The evaluation measurements are described in Supplementary Materials.

### 4.1 Performance on UniRef50 datasets

We first evaluated the proposed models on the UniRef50 datasets. In Forslund and Sonnhammer (2008), the FS model was compared with the best BLAST-based method, the Pfam2GO method and the MultiPfam2GO method. MultiPfam2GO is an extension of the Pfam2GO method that maps multiple InterPro domains to the Pfam domains. Both DRDO and DRDO-NB models were compared with these state-of-the-art methods on the same subsets of the UniRef50 dataset. Tables 1 and 2 show the comparison results on the two subsets. For both of the proposed models, the GO terms are ranked for each protein. The top ranked GO terms are selected until one of the following two conditions satisfied: (i) the probability of the next GO term has a difference that is bigger than 0.2 to that of the first GO term; (ii) the probability of the next GO term has a difference that is bigger than 0.08 to that of the previous one. The thresholds are selected as the ones that perform well for both the proposed models and the FS model on all the datasets.

**Table 1.** Summary of function prediction performance on the ‘curated annotations only’ subset of UniRef50

Dataset		Curated annotations only		
Dataset size		31,861 proteins		
	Sens.	Spec.	Prec.	MCC
Best BLAST	38.0	>99.9	42.4	0.40
Pfam2GO	5.5	>99.9	55.2	0.17
MultiPfam2GO	7.5	>99.9	52.3	0.20
FS model	25.9	>99.9	59.3	0.39
DRDO model	41.2	>99.9	88.0	0.56
DRDO-NB model	47.8	>99.9	75.8	0.54

All the values listed in the table, except for the MCC values, are percentages.

**Table 2.** Summary of function prediction performance on the ‘all’ subset of UniRef50

Dataset		All		
Dataset size		654,180 proteins		
	Sens.	Spec.	Prec.	MCC
Best BLAST	87.8	>99.9	82.1	0.85
Pfam2GO	53.3	>99.9	99.6	0.73
MultiPfam2GO	56.7	>99.9	99.4	0.75
FS model	69.1	>99.9	93.9	0.81
DRDO model	84.7	>99.9	89.2	0.84
DRDO-NB model	79.8	>99.9	91.3	0.87
DRDO model (PC)	72.5	>99.9	94.2	0.83
DRDO-NB model (PC)	75.8	>99.9	94.3	0.85

‘PC’ stands for the precision-controlled performance of the corresponding models, where the thresholds were set such that the DRDO model and the DRDO-NB model both had precision values greater or equal to that achieved by the FS model. All the values listed in the table, except for the MCC values, are percentages.

We observe that all methods tested here achieved an almost perfect specificity on both two datasets. As expected, when we considered only the proteins with curated annotations, the performance of all methods deteriorated, as shown in Table 1. The DRDO model and the DRDO-NB model significantly outperformed all the other prediction methods. To be more specific, by taking domain recurrence and order information into consideration, the DRDO model achieved an improvement of 59% on sensitivity and 48% on precision relative to the FS model. The two proposed models have comparable performance on this dataset. The DRDO-NB model gave higher sensitivity but lower precision values than the DRDO model did.

When all proteins in the UniRef50 dataset were considered, BLAST achieved the highest sensitivity, but the lowest precision, as shown in Table 2. Pfam2GO, on the other hand, had the highest precision, but the lowest sensitivity. The DRDO model and the DRDO-NB model provided good tradeoffs between sensitivity and precision. It can be seen in Table 2 that the FS model achieved a relatively high precision (93.9%) on the entire UniRef50 dataset. However, this is at the cost of missing true GO terms, which caused lower sensitivity (69.1%). One possible reason is that the FS model encoded only the distinct domain composition, but ignored the domain recurrence and order information. By using such information, the DRDO model achieved a precision value of 89.2% but much higher sensitivity (84.7%). The MCC value is also much higher than that of the FS model. We further controlled the thresholds for selecting GO terms such that our two models achieved at least the same precision as the FS model. The sensitivity values were then compared as shown in Table 2. Although the sensitivity for both proposed models decreased as a consequence of the higher precision, both proposed models had higher sensitivity than the FS model, demonstrating that our models performed better. However, we were not able to show explicitly that our models performed better than Pfam2Go and MultiPfam2Go, since we could not achieve the same precision as these two models. Indirect comparison via MCC suggested that our models overall could be more accurate than Pfam2Go and MultiPfam2Go.

### 4.2 Comparison on UniProtKB datasets

We further compared the proposed models with the FS model on a more up-to-date dataset, i.e. the recent UniProtKB dataset. The performance of the three methods on both UniProtKB/Swiss-Prot and the UniProtKB/TrEMBL datasets is shown in Table 3.

**Table 3.** Summary of function prediction performance on UniProtKB

Dataset	Swiss-Prot			TrEMBL		
Dataset size	497,872 proteins			10,168,218 proteins		
	Sens.	Prec.	MCC	Sens.	Prec.	MCC
FS model	12.3	45.4	0.29	55.0	93.1	0.71
DRDO model	20.5	75.2	0.37	67.2	89.2	0.77
DRDO-NB model	49.2	95.6	0.68	79.2	89.0	0.84

Comparison of the FS model, the DRDO model and the DRDO-NB model on the UniProtKB dataset. All values listed in the table, except the MCC values, are percentages.

**Fig. 4.**
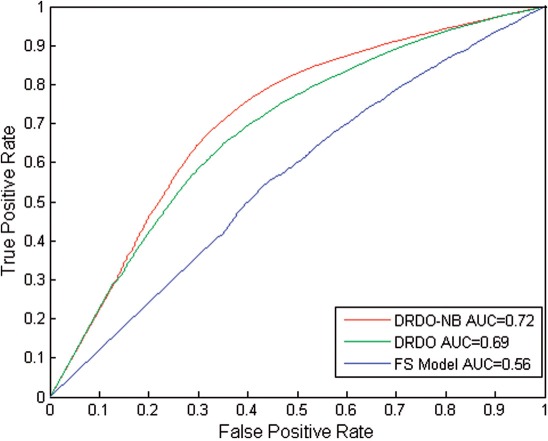
ROC curves for the FS model (blue), the DRDO model (green) and the DRDO-NB model (red) on the UniprotKB/TrEMBL 2011 database. The area under curve (AUC) for the three models are given in the legend

As shown in Table 3, the DRDO-NB model significantly outperformed the DRDO model in terms of sensitivity and MCC on both subsets. Both proposed methods, on the other hand, significantly outperformed the FS model. On the UniProtKB/TrEMBL dataset, the DRDO model outperformed the FS model by 22% in terms of the sensitivity, whereas the improvement was about 67% on the UniProtKB/Swiss-Prot dataset. Figure 4 also highlights the improvements of the proposed models over the FS model. The ROC curves were drawn by varying the threshold for choosing the GO terms along the sorted list as the predictions. These good improvements clearly demonstrate that the domain recurrence and domain order can enhance the accuracy of protein function prediction.

**Fig. 5.**
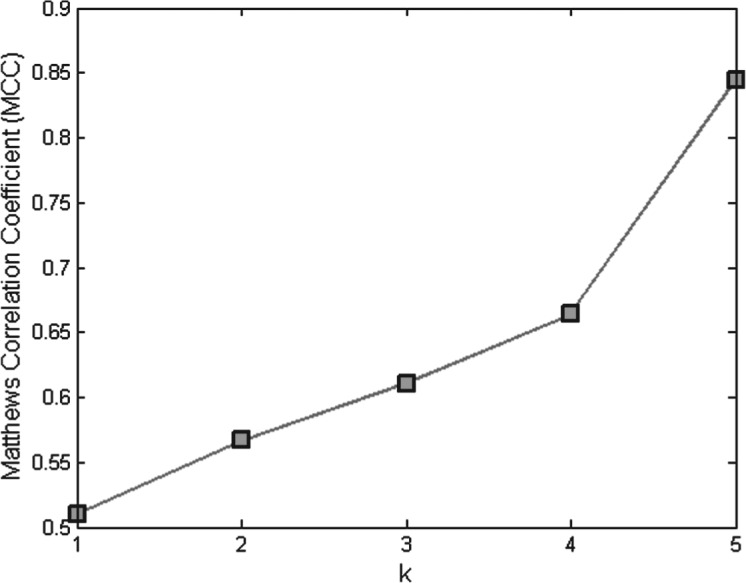
MCC curve of the DRDO-NB model on the UniprotKB/TrEMBL 2011 database, when different values for *k* are used. Only the proteins that have at least five domains are included in this experiment. Out of 10,168,218 proteins, 4,018,479 satisfy the requirements

Figure 5 shows the MCC of the DRDO-NB model when different values for *k* are used. All proteins in the UniprotKB/TrEMBL database that have at least five domains were used to assess the effect of the window size, *k*. Clearly, the performance of the model increases when the window size increases. This strongly supports our hypothesis that adding more domain information gives better results. Note that the MCC value when *k* is set to 5 is almost 0.84, which is slightly lower than the value in Table 3, which is the performance when the entire UniprotKB/TrEMBL database is used and all possible window sizes are enumerated. Therefore, better performance can be expected when more domain information is encoded.

By comparing the results in Table 3 to those in Tables 1 and 2, we can see that the performance of the FS and DRDO models decreases significantly on the UniProtKB data. In contrast, the performance of the DRDO-NB model is comparable on the ‘all’ subset of the UniRef50 data and the ‘UniProtKB/TrEMBL’ subset of the UniProtKB data. When only the proteins with curated annotations are considered, the performance of the DRDO-NB model on the ‘UniProtKB/Swiss-Prot’ subset actually significantly improves over that on the ‘curated annotations only’ subset, suggesting that the DRDO-NB model is more robust than the other models.

### 4.3 Limitations

Both of our two models predict the GO terms for query proteins by assuming the input of GO terms for each domain in the protein. Although our models demonstrate significant improvements over the state-of-the-art methods, the sensitivity and precision is still not perfect. One of the main limitations in our method is that we rely on the information contained in some databases and these may not contain complete information or their records could be erroneous. In other words, the TrEMBL database has partial association of GO-terms to proteins and also has many wrong annotations which affect the accuracy of our approach greatly. On the other hand, the curated database Swiss-Prot is much more accurate than the TrEMBL, but it also contains partial GO annotation and is considerably smaller. This also affects the accuracy of our approach. The following is a detailed description of the source of inaccuracies in our implemented method: the main source of false positives is the GO terms that appear frequently in the subsets of the domains of the query protein, but are not associated with the query protein. On the other hand, there are two main sources for the false negatives. The first is that given a query protein, SwissPfam and UniProtKB databases are used to extract domain architecture and the corresponding GO terms for each domain; if any GO term for the query protein is missed in these databases, our methods cannot predict this GO term. The second is that if a GO term appears very frequently in single domains of the query protein, the threshold in our models to select GO terms can become high which results in falsely filtered out GO terms. Furthermore, including more subsets in our models can boost performance as suggested by Figure 5. However, in practice, we could not include all the possible combinations of domains since this is computationally infeasible. Finally, our models are domain-based approaches and thus cannot distinguish between proteins that have the same domain architectures but different amino acid sequences.

## 5 DISCUSSION

The main problem with sequence-based function predictors is that the number of false positives is high. Domain-based predictors, on the other hand, can achieve much higher precision. Therefore, an ideal protein function annotation predictor should be able to encode both the amino acid information and the domain information. We are currently exploring how to use this sequence information in combination with our proposed models. Another possible improvement to our models can be achieved by using the spatial positions of the domains with predicted or native tertiary structural information. In addition, we are also trying to consider the hierarchical nature of GO terms as a directed acyclic graph (de Lima Morais *et al*., 2011) and checking the effects of considering the GO terms from the MF and biological process (BP) separately, since they are quite different in describing functional signals inherited in domains. Furthermore, the overlap between the predictions made by DRDO and DRDO-NB is about 70% (as shown in Supplementary Table S4). It is thus possible to combine the prediction results from DRDO and DRDO-NB to further enhance the accuracy.

As shown in Figure 2, most of the existing proteins have fewer than 10 domains, whereas the largest number of domains found so far for a protein is less than 60. This property enables efficient enumeration as used in our methods and other domain-based predictors.

The main focus of this article is to demonstrate that domain recurrence and order is important for predicting protein functions. Previous methods showed that considering domain architecture improves prediction over conventional sequence similarity-based methods. Here, we show that domain recurrence and order further enhance protein function inference.
